# Depression in Polycystic Ovary Syndrome: A Systematic Review and Meta-Analysis

**DOI:** 10.3390/jcm12206446

**Published:** 2023-10-10

**Authors:** Paweł Dybciak, Dorota Raczkiewicz, Ewa Humeniuk, Tomasz Powrózek, Mariusz Gujski, Teresa Małecka-Massalska, Artur Wdowiak, Iwona Bojar

**Affiliations:** 1 Centre of Postgraduate Medical Education, Marymoncka Street 99/103, 01-813 Warsaw, Poland; paweldybciak@gmail.com; 2 Department of Medical Statistics, School of Public Health, Centre of Postgraduate Medical Education, Kleczewska 61/63 Street, 01-826 Warsaw, Poland; 3 Chair and Department of Psychology, Faculty of Medical Sciences, Medical University of Lublin, Chodzki 7 Street, 20-400 Lublin, Poland; ewa.humeniuk1@gmail.com; 4 Department of Human Physiology, Medical University of Lublin, Radziwiłłowska 11 Street, 20-080 Lublin, Poland; tomaszpowrozek@gmail.com (T.P.); teresa.malecka-massalska@umlub.pl (T.M.-M.); 5 Department of Public Health, Medical University of Warsaw, Jana Nielubowicza 5 Street, Blok F, 02-097 Warsaw, Poland; mariusz.gujski@wum.edu.pl; 6 Chair of Obstetrics and Gynecology, Faculty of Health Sciences, Medical University of Lublin, Staszica 4-6 Street, 20-081 Lublin, Poland; wdowiakartur@gmail.com; 7 Department of Women’s Health, Institute of Rural Health, Jaczewskiego 2 Street, 20-090 Lublin, Poland; iwonabojar75@gmail.com

**Keywords:** polycystic ovary syndrome, depression, mental health

## Abstract

Polycystic ovary syndrome (PCOS) is an endocrine disorder with a broad spectrum of clinical symptoms. Some of the serious complications of PCOS are mental disorders including depression. Therefore, the aim of the meta-analysis was to determine the prevalence, mean level, standardized mean difference and probability of depression based on the research conducted with the Hospital Anxiety and Depression Scale (HADS). A systematic literature search was performed using the following databases: PubMed, EMBASE, Scopus, ClinicalTrials.gov and Google for research published until January 2023. The meta-analysis was conducted on a group of 4002 patients obtained from 19 studies, which met the inclusion criteria (adult pre-menopausal women diagnosed with PCOS, papers on the prevalence of depression or the HADS scoring). According to the research performed, the mean prevalence of depression was 31% (I2 = 93%; *p* < 0.001), whereas the mean HADS depression score in patients with PCOS was 6.31 (I2 = 93%; *p* < 0.001). The standardized difference of mean depression scores was SMD = 0.421 (95% confidence interval = 0.17–0.68, I2 = 67%). The overall probability of depression in PCOS patients was more than 2.5-fold higher than in healthy women ((RR: 2.58), confidence interval [1.38–4.85]; I2 = 90%, *p* < 0.001). The research results imply an increased risk of depressive symptoms in women with PCOS.

## 1. Introduction

Polycystic ovary syndrome (PCOS) is one of the most common endocrine disorders. Its prevalence, depending on the diagnostic criteria applied, ranges from 2.2% to 26.7% worldwide [1,2,3].

The most widely used definition of PCOS is the one according to the Rotterdam consensus criteria, which determines that the syndrome can be diagnosed if at least two out of the three symptoms occur: clinical and/or biochemical hyperandrogenism, ovulatory dysfunction or the presence of an ovarian cyst in ultrasound examination [4,5]. It is a heterogeneous disorder with a broad spectrum of clinical symptoms affecting various systems and organs [6,7].

The main clinical symptoms include irregular menstruation or lack of menstruation cycles and/or infertility [8]; acne vulgaris, androgenic alopecia, hirsutism [9]; dyslipidemia and endothelial dysfunction markers [10]; hyperinsulinemia, insulin resistance and increase in BMI [11,12].

PCOS is a chronic disease and available treatments can only help to control the signs and symptoms and reduce the risk of complications [13].

Some of the serious consequences of PCOS are mental disorders including depressive ones. In particular, indicators of depression and anxiety in women with PCOS are well documented in the literature [14,15,16,17,18]. The prevalence of depression in women with PCOS is high and ranges from 16% to 55% [19,20]. The most frequently observed signs and symptoms of depression in women with PCOS are fatigue, sleep disorders and low mood [13]. Suicidal thoughts and attempts are less frequently observed, although their probability was seven-fold higher than in the control group [21].

Depressive disorders are defined as deep and persistent lowered mood. According to the data compiled by the World Health Organization (WHO), a total of 322 million people suffered from depressive disorders worldwide in 2015. The prevalence of depression in the general population ranged from 2.6% to 5.9% and the number of depression sufferers increased by 18.4% between 2005 and 2015 [22,23]. The risk factors for the development of depression include both biological (genetic, hormonal, biochemical, proinflammatory and medications and somatic diseases) and individual ones (personality, life history) as well as psychosocial ones (life situation, traumatic and stressful events) [24,25,26].

According to the literature review conducted by Accortt et al. 2008, women suffer from depression almost twice as often as men [27]. In this context, all the studies concerned with depression in women, particularly the ones suffering from chronic diseases, appear to be highly justified. Recent systematic reviews and meta-analyses have confirmed that women with PCOS presented a three-fold higher probability of depressive symptoms compared with the women of the same age from the control group [14,28,29,30,31,32]. In another meta-analysis, it was depicted that the overall prevalence of depression was 36.6% and higher probability of moderate and severe depressive symptoms was observed in patients with PCOS compared to controls (OR = 4.18; 95%CI: 2.68–6.52) [29].

Research results on the causes of higher prevalence of depression in women with PCOS are ambiguous. Depressive disorders may result from the aggravating symptoms of PCOS, hormonal changes or a combination of the two factors [20]. A higher level of depression was found in women with infertility [15,16,20]. Numerous studies depicted that the level of depression was related to obesity and dissatisfaction with the appearance of their body [29,33]. In contrast, other studies showed a relationship between high serum androgen level and depression [34]. It was also proved that women with hirsutism and acne [29] as well as insulin resistance and type 2 diabetes were more prone to depression [29,35]. Although the exact etiology of the link between PCOS symptoms and depression is still a matter of debate, a recent article attempted to synthesize the etiological factors mentioned above in connection with those relating to neurotransmitters [36].

Taking into account the results of previous studies and the fact that mood has a significant effect on the quality of life but also the ability to undertake actions aimed at coping with this chronic disease, all attempts to broaden the knowledge on the risk of mood disorders in women with PCOS appear to be of great importance.

According to the studies conducted so far, the prevalence of depression and its levels were varied, which, according to some researchers, can result from the fact that various tools were used to diagnose depression [32,34,37]. Therefore, the aim of the meta-analysis is determination of:

1. Prevalence of depression;
2. Mean level of depression;
3. Standardized difference in mean scores in groups of women with PCOS and healthy ones;
4. Probability of depression based on studies performed using the Hospital Anxiety and Depression Scale (HADS).

The HADS is a reliable screening instrument for clinically significant anxiety and depression in patients presenting to medical clinics. It is also an essential measure of the severity of mood disorders. The questionnaire comprises fourteen questions, including seven items for the assessment of anxiety (HADS-A) and seven for depression (HADS-D). A score of 0–7 on the HADS-D scale is considered normal, 8–10 suggests mild changes and a score of 11 or higher suggests the probable presence of depressive disorders. The HADS showed good reliability for depression (a = 0.82) in an extensive validation study [38,39].

It has been assumed that the use of the HADS to diagnose depression would provide greater methodological consistency and the results would be more comparable and probably more reliable than in the case of previous meta-analyses which utilized various scales.

## 2. Materials and Methods

### 2.1. Search Methodology

The study was designed according to Preferred Reporting Items for Systematic Re-views and Meta-Analyses (PRISMA) [40]. A systematic literature search was conducted using four databases: PubMed (until January 2023), EMBASE (until January 2023), Scopus (until January 2023), ClinicalTrials.gov. We used Google searches as a supplement for the basic search of the most commonly used databases. This method of data search has been used in other meta-analyses and has been found valuable. Google provides a quick algorithm for searching information even if the keywords used by several authors in their papers were not detailed. Sometimes, sources available in PubMed or Scopus are restricted only to the title or abstract of the study. Thus, Google search allowed us to reach the papers from other sources and eventually include them in the meta-analysis. The literature search was limited to 2009 to the end of January 2023.

The following approach to the literature search was applied with the use of appropriate keywords: “polycystic ovary syndrome”, “PCOS”, “mental disorders”, “depression”, “depressive disorder”, “HADS”. Moreover, keywords were entered into the keyword search tool as follows: (“polycystic ovary syndrome” OR “PCOS”) and (“mental disorders” OR “depression” OR “depressive disorders” OR “HADS”). In addition to the abovementioned searching strategy, PubMed was searched using MeSH terms such as: polycystic ovary syndrome (MeSH: “ovary syndrome, polycystic”, “syndrome, polycystic ovary”, “polycystic ovary syndrome”), depression (MeSH: “depressive symptoms”, “emotional depression”, “symptoms, depressive”) (all boxes).

The listed MeSH terms used in the data search were used separately and were also combined with each other.

Our research was based on the PRISMA guidelines from http://www.prisma-statement.org and https://www.ncbi.nlm.nih.gov/pmc/articles/PMC6461330/. “URL (accessed on 4 August 2023)”.

The application for the study was registered in PROSPERO, number CDR42023432656.

### 2.2. Eligibility

Both the inclusion and exclusion criteria were used for the systematic review. The following inclusion criteria were applied:

1. The study included adult women aged over 18 years;
2. The study included pre-menopausal women;
3. The study included women who were diagnosed with polycystic ovary syndrome based on the criteria developed by the National Institutes of Health (NIH), the Rotterdam consensus or others [41];
4. It was possible to extract from the text the information about the prevalence of depressive symptoms in the study group;
5. It was possible to extract the HADS-D score from the text;
6. The study group included 30 patients or more;
7. Abstracts were qualified if they contained all the needed information mentioned above.

The following exclusion criteria were defined:

1. Review/case report/meta-analysis/systemic review;
2. Studies not written in English;
3. Studies in which adolescent or post-menopausal women were recruited;
4. Studies that did not assess the level and prevalence of mental disorders using the HADS;
5. Studies that assessed the effectiveness of a drug and one of the indicators was the level of depression;
6. Studies in which depressive disorders were assessed only in terms of obesity, infertility, hirsutism or other symptoms of PCOS;
7. Studies that included pregnant patients with PCOS.

No restrictions concerned with the place where the research was conducted were imposed. The search and selection of studies were organized following the Population, Intervention Comparison, Outcomes and Study Design (PICOS) strategy (Table 1).

### 2.3. Data Analysis

MetaXL (EpiGear, Sunrise Beach, Australia) software version 5.3 was applied to analyze data and generate graphs. Cochrane’s Q Test (Q) with the I2 test was used to assess heterogeneity or the degree of variability in the true effect size estimated in the studies. A random effects model was used to present heterogeneity. The random effects model automatically handles the variability estimation for all random effects in the model. Moreover, the random effects model assumes that the true effect could vary from study to study due to the differences (heterogeneity) among studies. Based on the abovementioned, we used this strategy for data analysis. The following criteria were used to assess the level of heterogeneity: I2 ranging from 0–40% means a lack of heterogeneity/rejected heterogeneity, I2 > 40–70% refers to substantial heterogeneity and results of I2 over 70% indicate considerable heterogeneity. The standardized mean difference (SMD; Hedges’g) was used to measure the difference between the HADS means in patients with PCOS and those from the control group. SMDs of 0.2–0.5, 0.5–0.8 or >0.8 are considered as small, medium or large effects. A risk ratio (RR) over 1.0 is considered as a higher collective risk, whereas RR below 1.0 means low collective risk of mental disorder in the populations researched. Funnel plots followed by Luis Furuya-Kanamori index (LFK index) was used to check the presence of publication bias. The LFK index was interpreted in the following manner: LFK index below 1 means lack of asymmetry, LFK index between 1 and 2 reflects minor asymmetry, whereas LFK index over 2 indicates major asymmetry. In all the analyses, values of *p* < 0.05 were considered as statistically significant. For multiple comparisons, we checked Cronbach’s alpha values with K-corrected alpha values, including Kristof’s correction for sample size. The corrected values did not significantly vary from those previously obtained.

### 2.4. Search Results

The systematic review of literature was completed on 31 January 2023 followed by the assessment of eligibility of article titles, keywords and abstracts. In the course of the first selection, two independent double-blind researchers reviewed 1092 studies sourced and then removed 469 duplicates. A total of ten questionable papers were discussed and a joint decision was made after negotiations (kappa statistics, 0.96). Since 387 studies did not meet the PRISMA criteria, 82 eligible ones were ultimately selected. Then, 63 studies were excluded because they: involved adolescent or post-menopausal female patients (14 studies), had insufficient data, used different measurement tools or the necessary data could not be extracted (19 papers), depression was analyzed in a different context, e.g., treatment (13 studies), measurement tools other than the HADS were used (17 studies). This reduced the total number of eligible studies to 19.

Where possible, the following data were extracted from each paper: diagnostic criteria for PCOS and whether it was confirmed in an examination or reported by the patients, all the characteristics by which PCOS patients and controls were matched. Groups were most frequently matched based on the following variables: age, ethnicity, level of education, place of residence, employment, marital status, number of children, BMI.

**Figure 1:**
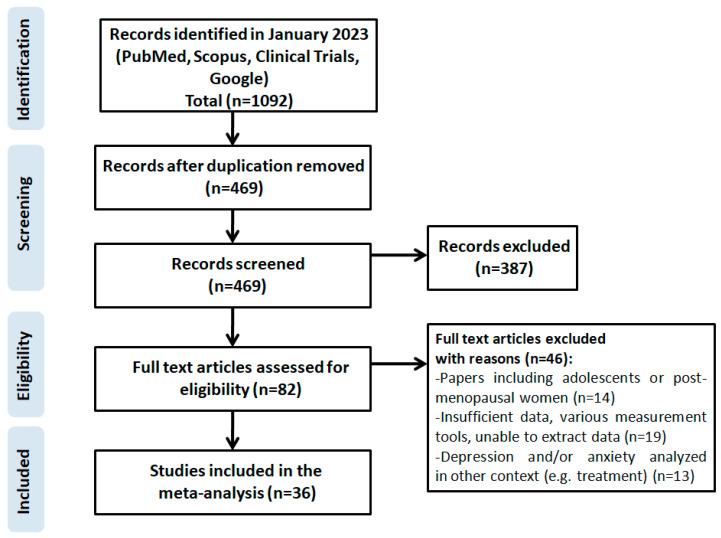
Scheme of research methodology for the systematic review based on the PRISMA guidelines.

The following information was obtained from the eligible studies: the first author, year of publication, country where the research was conducted, the size of the research sample, prevalence of depression and/or mean HADS score. The detailed strategy used to identify, search for and select the literature is depicted in Figure 1.

## 3. Results

**Figure 2:**
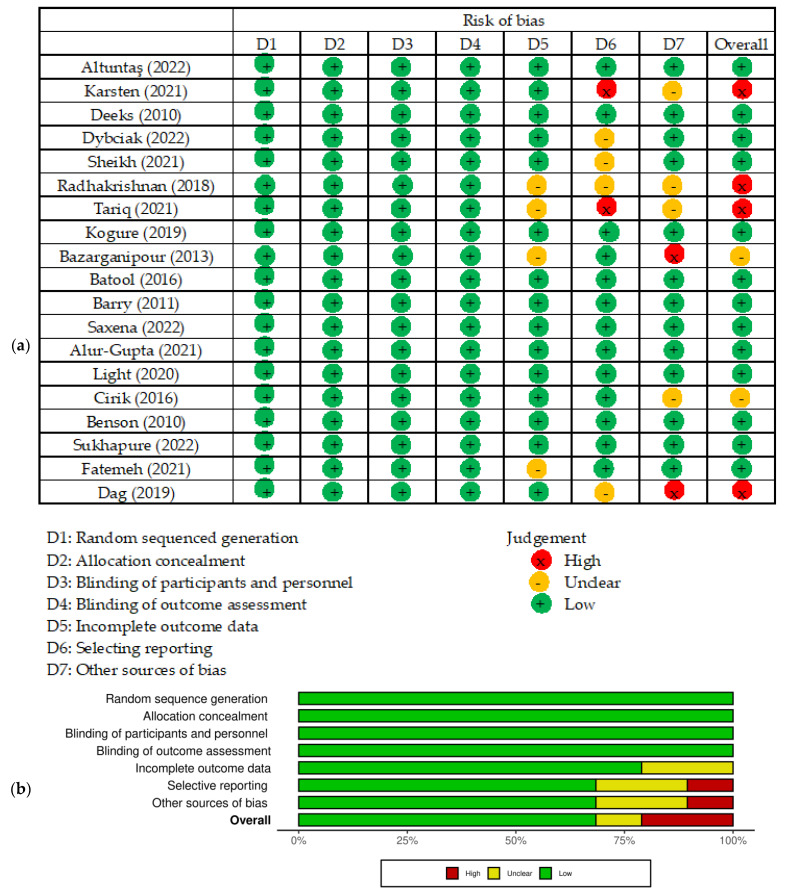
Risk of bias of studies included in the meta-analysis. (**a**) Risk of bias summary: review of the authors’ own judgments about each risk of bias item for each included study. (**b**) Risk of bias graph: review the authors’ own judgments about each risk of bias item presented as percentages across all included studies. Figure generated by Cochrane Risk of Bias 2 (RoB 2) tool, [6,7,15,16,17,19,20,33,34,35,37,42,43,44,45,46,47,48,49].

The results of the risk of bias assessment for each study included in the meta-analysis conducted with Cochrane Risk of Bias 2 (RoB 2) tool are presented in Figure 2. Regarding the selection of participants, 13 studies were judged as having a low risk of bias, and 6 studies having a high or unclear risk of bias due to unclear confirmation of the study site.

**Figure 3:**
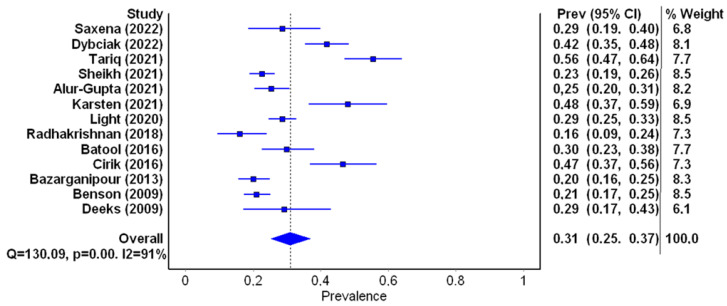
Forest plot of the meta-analyzed prevalence of depression in PCOS patients (figure generated by MetaXL (EpiGear, Sunrise Beach, Australia) software version 5.3), [7,15,16,17,19,20,34,35,40,42,44,45,46].

General characteristics of the studies included in the meta-analysis are summarized in Table 2. A total of 13 studies were qualified for the first part of the meta-analysis. They all met the inclusion criteria and contained data on the number of patients with a score on the depression scale of ≥8 points, which gave 2903 patients. Six studies were from Asian countries, five from European countries, one from the USA and one from Australia. The prevalence of depression ranged from 16% to 55.6% (mean 31%). The lowest and highest depression prevalence scores were observed in South Asia, i.e., India [19] and Pakistan [20]. The prevalence of depression in selected studies is presented in Table 3. A random effects model depicted a considerable heterogeneity of the research results (I2 = 93%; *p* < 0.001, Figure 3).

**Figure 4:**
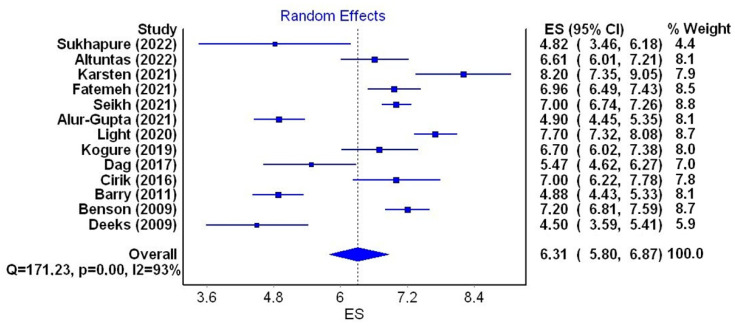
Forest plot of the meta-analyzed mean depression scores in PCOS patients (figure generated by MetaXL (EpiGear, Sunrise Beach, Australia) software version 5.3) [6,7,15,17,33,37,43,44,45,46,47,48,49].

For the next stage of the meta-analysis, a total of 13 studies were qualified. They met the inclusion criteria and contained data on the mean HADS score, which amounted to 2619 women aged from 22.69 to 34.1 years. In one of the studies, the mean age was not stated [17]; in another, the median age was given [43]. The mean depression score ranged from 4.5 to 8.2 (mean 6.31). The lowest score was obtained by patients from Australia (4.5) and New Zealand (4.82), the highest was observed in patients from the Netherlands (8.2), the UK(7.70) and Brazil (7.2). Mean HADS-D scores obtained by patients with PCOS are presented in Table 4. A random effects model depicted considerable heterogeneity of the research results (I2 = 93%; *p* < 0.001) (Figure 4).

**Figure 5:**
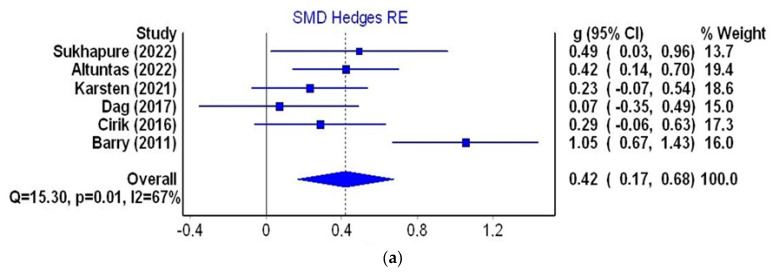

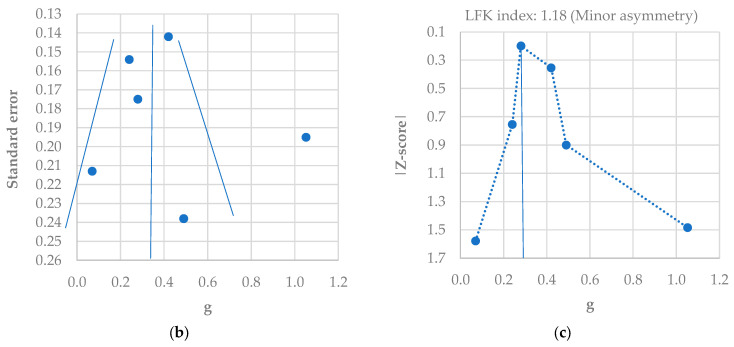
Meta-analysis using a random effects model for the SMD of the HADS-D score between control and PCOS individuals (**a**); funnel plot of SMD for depression meta-analysis (**b**); Doi plot and LFK index for the detection of publication bias (**c**). Figures generated by MetaXL (EpiGear, Sunrise Beach, Australia) software version 5.3 [6,7,37,45,47,49].

Six studies (three from Turkey and one each from the UK, the Netherlands and New Zealand) were qualified for the next stage of the meta-analysis. They met the inclusion criteria and contained data on the mean HADS score in the group of women with PCOS and in the control group. The group of women with PCOS comprised 503 patients and the control group consisted of 350 women. The mean age of women with PCOS ranged from 22.69 to 34.1 years and in the control group it was from 21.34 to 35.12 years. The smallest standardized mean difference (SMD = 0.070, confidence interval = 0.35 to 0.49) was observed in a study of 53 Turkish patients [49], and the highest (SMD = 1.052, confidence interval from 0.67 to 1.43) in a group of 76 patients from the UK [37]. The cumulative SMD between the mean level of depression in the PCOS group and the control group was 0.421 (95% confidence interval from 0.17 to 0.68, I2 = 67%), indicating a small overall effects size with substantial heterogeneity (Figure 5a). Detailed data on the HADS-D values in both groups and the SMD (Hedge’s g) are depicted in Table 5.

A slight asymmetry in the funnel plot was found (Figure 5b), which was confirmed by an LFK score of 1.18 (minor asymmetry) (Figure 5c). This means that there was no significant publication bias in the six studies included.

**Figure 6:**
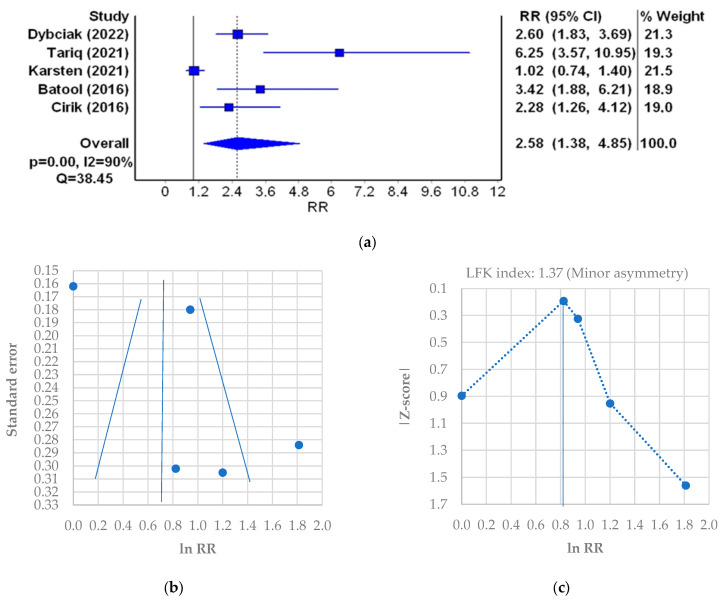
Relative risk (RR) meta-analysis plot (random effects model): probability of the incidence of depression in PCOS patients (**a**); funnel plot of RR for depression meta-analysis (**b**); Doi plot and LFK index for the detection of publication bias (**c**). Figures generated by MetaXL (EpiGear, Sunrise Beach, Australia) software version 5.3 [7,16,20,35,45].

In the next stage of the meta-analysis, the probability of depression in PCOS patients was assessed (Table 6). Five studies that met inclusion criteria and contained the data on the number of PCOS patients and controls with a depression score of ≥8 points were qualified, which resulted in 290 PCOS patients and 113 controls. The studies included were of similar weight. The lowest probability of depression (RR [95%CI] = 1.02 [0.74–1.40]) was found in the study of 35 patients from the UK [7]. The highest probability of depression (RR [95%CI] = 6.25 [3.57–10.95]) was observed in the study of 75 patients from Pakistan [20]. The cumulative probability of depression in the PCOS patients is more than 2.5-fold higher than in healthy women (RR [95%CI] = 2.58 [1.38–4.85], I2 = 90%, *p* < 0.001) (Figure 6a). A minor asymmetry was found in the funnel plot (Figure 6b) which was confirmed by the LFK score of 1.37 (minor asymmetry) (Figure 6c).

## 4. Discussion

The aim of the meta-analysis was to determine: prevalence of depression, mean level of depression, standardized mean difference and probability of depression based on the research conducted using the Hospital Anxiety and Depression Scale (HADS). The presented meta-analysis was based on 4002 patients with PCOS obtained from 19 studies that met the inclusion criteria and originated from different countries (4 UK, 3 Turkey, 2 India, 2 Iran, 2 Pakistan, and 1 each from Australia, Brazil, the Netherlands, New Zealand, Poland and the USA). The result of the I2 test for the majority of the analyses performed showed considerable heterogeneity, which is why a random effects model was used. The highest heterogeneity (I2 = 93%) concerned studies that determined the mean level of depression in PCOS patients, the lowest (I2 = 67%) concerned studies based on which the standardized mean difference of the results in the group of PCOS patients and controls was calculated.

On the basis of the meta-analysis of 13 studies (2903 people) regarding the prevalence of depression of ≥8 points on the HADS, it was found that it ranged from 16% to 55.6%, with a mean value of 31% (I2 = 93%, *p* < 0.001). Moreover, the lowest prevalence of depression was noted in India [19] and the highest was presented by the women in Pakistan [20]. The result obtained is the lowest compared to the meta-analyses already performed, where prevalence of depression was 36.6%(IQR 22.3–50.0%) [29] and 42% (95%CI 33–52%) [31]. The difference might be due to the fact that these meta-analyses included studies that utilized various diagnostic instruments. It appears that the tool used to measure depression may affect the frequency of diagnosis of depressive disorders.

The meta-analysis showed that the mean HADS score in PCOS patients (13 studies, 2619 women aged from 22.69 to 34.1 years) was 6.31 (I2 = 93%, *p* < 0.001). Scores ranged from 4.5 (Australia) to 8.2 (UK) [7,15]. This result cannot refer to the ones obtained in previous meta-analyses as they do not contain such data [14,28,29,31,32,34,37].

The standardized mean difference of the HADS scores (6 studies, 503 PCOS patients and 350 controls) in the present study was SMD = 0.421 (95%CI 0.17–0.68, I2 = 67%), which is due to a small overall value effect with substantial heterogeneity and minor publication bias of the studies included (LFK = 1.18).

This result is significantly lower compared to previous meta-analyses. The highest standardized mean difference (0.82, 95%CI 0.73–0.92) was obtained in the meta-analysis by Barry, Kuczmierczyk et al., 2011, whereas Yin et al. obtained a result of 0.64, 95%CI 0.50–0.78 [32,34]. Nevertheless, in the meta-analysis by Veltman-Verhulst et al., SMD = 0.60 (95%CI 0.47–0.73) [50]. It is worth emphasizing, however, that all the cited papers were based on studies using various methods of diagnosing depression.

The assessment of probability of depression in PCOS patients compared with the control group showed that the overall probability of depression in PCOS patients based on mean scores is more than 2.5-fold higher than in healthy women (RR = 2.58, 95%CI 1.38–4.85, *p* < 0.001, with considerable heterogeneity of 90%) and minor publications bias (LFK = 1.37).

Similar results to those of Wang et al. 2021 were obtained, where women with PCOS were twice as likely to be diagnosed with depression than controls (OR = 2.098; 95%CI 1.411–3.119; four studies) [31], and Brutocao et al. 2018, where PCOS was related to 2.79-fold higher probability of diagnosing clinical depression (OR = 2.79, 95%CI 2.23–3.50) [14] compared to the control group.

Although the evidence for higher prevalence of depression in PCOS is convincing, none of the meta-analyses provides a definitive explanation of the cause of this relationship [29,31,34,37]. Researchers have aimed to explain by which mechanisms PCOS is linked to depression. First of all, the symptoms of PCOS cause psychological stress in patients [51] and as the research shows, hypercortisolism and hyperactivity of the HPA axis play a pivotal role in the development of depression [52]. Evidence from numerous studies shows that inflammatory factors play a key role in the genesis of mental disorders [53], and PCOS has been shown to be a proinflammatory condition [54]. On the other hand, many of the specific metabolic changes associated with PCOS, namely insulin resistance, obesity and androgen excess, have also been observed in patients suffering from affective disorders [55]. Thus, the signs and symptoms of depression and PCOS overlap [56]. Therefore, well-designed studies assessing the effect of treating these factors on the depressive symptoms in PCOS women are required.

The strengths of the study are that it included only those studies in which one method of diagnosing depression (the HADS) was utilized. To the best of our knowledge, no such a meta-analysis has been conducted so far. All the previous ones were based on diagnosis using various methods such as the Beck Depression Inventory (BDI), the 12-item General Health Questionnaire (GHQ-12) and the Depression Anxiety Stress Scale (DASS). It is highlighted that this might have been one of the sources of heterogeneity of results [32,34]. Furthermore, in the subsequent steps of the meta-analysis, studies both with and without control groups were included. It enabled determination of the prevalence and mean level of depression in the group of women with PCOS and, based on the research with the control group, determination of the standardized mean difference and probability of depression.

The study also has some limitations. All the studies included in the meta-analysis were cross-sectional, therefore it can only be hypothesized that diagnosis of PCOS is preceded by the diagnosis of depression and anxiety. The main limitation of the paper is the high heterogeneity of the studies. Secondly, the study included only published articles, without other types of papers, which might have resulted in publication bias. Moreover, only studies written in English were included. Furthermore, the impact of such factors as infertility, androgen levels, BMI or duration of the disease, which may play a significant role in mental health, was not studied. Unfortunately, these data, when combined with other inclusion criteria applied, were impossible to extract in the majority of cases.

## 5. Conclusions

Our findings suggest an increased risk of depressive symptoms in the population of women with PCOS and thus highlight the importance of screening examinations and appropriate follow-up in this population. The data obtained confirm the need to implement guidelines into routine practice, according to which women with PCOS should undergo mental health screening and long-term follow-up [57,58].

The worrying mental health situation of women with PCOS highlights the urgent need for developing psychological healthcare interventions. Psychoeducation on PCOS is vital in this area. It should be conducted by doctors or other healthcare professionals, given that the disease is still not well known among patients [59] and medical professionals [60]. It is crucial that women with PCOS are aware of the threats that the disease poses to their mental health and how to seek professional help if necessary. Moreover, it should be emphasized that every consultation should be an opportunity not only to assess the clinical aspects of PCOS but also the clinical symptoms of depression, which can be effectively treated. Talking to a patient provides an opportunity to discuss important issues resulting from PCOS, such as: marital, family or social problems, low quality of life, sexual dysfunctions, low self-esteem. It also seems essential to use well-proven tools that give a chance to make an accurate diagnosis.

Further research on the most effective methods of psychosocial interventions for women with PCOS should be conducted. Even though various methods of therapy aimed at improvement of mental health and quality of life in women with PCOS, such as cognitive behavioral therapy, are used, the interventions are still limited and their effectiveness is unknown [61].

## Figures and Tables

**Table 1:** Population, Intervention, Comparison, Outcomes and Study Design (PICOS) for the study.

PICOS	Application of the Criteria on the Present Study
Population	Adult and pre-menopausal women with PCOS
Intervention	None
Comparison	Studies included both with and without control group (healthy women)
Outcomes	Group size; depression measured by HADS-D score; relative risk of depression
Study Design	Case–control, cross-sectional, prospective and retrospective studies written in English

**Table 2:** Characteristics of the studies included in the meta-analysis.

Study (Year)	Country	Number of Patients		PCOS Patients’ Age
		PCOS	Control	
Altuntaş (2022) [6]	Turkey	167	73	25.87 ± 5.64
Karsten (2021) [7]	Netherlands	73	100	34.1 ± 4.1
Deeks (2010) [15]	Australia	48	-	32.5 ± 7.98
Dybciak (2022) [16]	Poland	230	199	20–40
Sheikh (2021) [17]	United Kingdom	502	-	-
Radhakrishnan (2018) [19]	India	100	-	16–36
Tariq (2021) [20]	Pakistan	135	135	15–45
Kogure (2019) [33]	Brazil	120	-	28.8 ± 5.2
Bazarganipour (2013) [34]	Iran	300	-	26.56 ± 4.44
Batool (2016) [35]	Pakistan	137	137	25.0 ± 8.6
Barry (2011) [37]	United Kingdom	76	49	28.8 ± 4.81
Saxena (2022) [42]	India	70	-	25.7 ± 7.6
Alur-Gupta (2021) [43]	United States of America	272	-	Median 28.9
Light (2020) [44]	United Kingdom	487	-	30.81 ± 7.65
Cirik (2016) [45]	Turkey	101	49	24.44 ± 4.23
Benson (2010) [46]	Germany	448	-	29.6 ± 5.5
Sukhapure (2022) [47]	New Zealand	33	41	29.24 ± 6.81
Fatemeh (2021) [48]	Iran	239	-	30.92 ± 5.49
Dag (2019) [49]	Turkey	53	38	22.69 ± 4.54

**Table 3:** Prevalence of depression in patients with PCOS.

Study (Year)	Number of Patients and Country	Age (Mean ± SD Years)	Depression (%)	Citation
Karsten (2021)	73 The Netherlands	34.1 ± 4.1	47.9% 35/73	[7]
Deeks (2010)	48 Australia	32.5 ± 7.98	29.2% 14/48	[15]
Dybciak (2022)	230 Poland	Not specified	41.7% 96/230	[16]
Sheikh (2021)	502 UK	Not specified	22.5% 113/502	[17]
Radhakrishnan (2018)	100 India	Not specified	16% 16/100	[19]
Tariq (2021)	135 Pakistan	Not specified	55.6% 75/135	[20]
Bazarganipour (2013)	300 Iran	26.56 ± 4.44	20% 60/300	[34]
Batool (2016)	137 Pakistan	25.0 ± 8.6	30% 41/137	[35]
Alur-Gupta (2021)	272 USA	Median 28.9	25.4% 69/272	[40]
Saxena (2022)	70 India	Not specified	28.5% 20/70	[42]
Light (2020)	487 UK	30.81 ± 7.65	23.8% 116/487	[44]
Cirik (2016)	101Turkey	24.44 ± 4.23	46.5% 47/101	[45]
Benson (2010)	448 Germany	29.6 ± 5.5	21% 94/448	[46]

**Table 4:** Mean HADS-D scores in PCOS patients.

Study (Year)	Number of Patients/Country	Age (Mean ± SD Years)	HADS-D Score (Mean ± SD)	Citation
Altuntaş (2022)	167 Turkey	25.87 ± 5.64	6.61 ± 3.99	[6]
Karsten (2021)	73 The Netherlands	34.1 ± 4.1	8.2 ± 3.7	[7]
Deeks (2010)	48 Australia	32.5 ± 7.98	4.5 ± 3.2	[15]
Sheikh (2021)	502 UK	Not specified	7 ± 3.0	[17]
Kogure (2019)	120 Brazil	28.8 ± 5.2	6.7 ± 3.8	[33]
Barry (2011)	487 UK	28.8 ± 4.81	4.88 ± 1.98	[37]
Alur-Gupta (2021)	272 USA	Median 28.9	4.9 ± 3.8	[43]
Light (2020)	487 UK	30.81 ± 7.65	7.7 ± 4.3	[44]
Cirik (2016)	101 Turkey	24.44 ± 4.23	7.0 ± 4.0	[45]
Benson (2009)	120 Brazil	29.6 ± 5.5	7.2 ± 4.2	[46]
Sukhapure (2022)	33 New Zealand	29.24 ± 6.81	4.82 ± 4.0	[47]
Fatemeh (2021)	239 Iran	30.92 ± 5.49	6.96 ± 3.73	[48]
Dag (2017)	53 Turkey	22.69 ± 4.54	5.47 ± 2.97	[49]

**Table 5:** Standardized mean difference of the HADS scores for depression in PCOS patients and healthy controls.

Study (Year) Country	Number of Cases	Age (Years) (Mean ± SD)	HADS-D Score (Mean ± SD)	SMD (Hedge’s g)	Citation
Altuntaş (2022) Turkey	Study group—167	25.87 ± 5.64	6.61 ± 3.99	0.422	[6]
	Control—73	27.25 ± 5.85	4.99 ± 3.41		
Karsten (2021) Netherlands	Study group—73	34.1 ± 4.1	8.2 ± 3.7	0.233	[7]
	Control—100	35.1 ± 3.7	7.4 ± 3.2		
Barry (2011) UK	Study group—76	28.8 ± 4.81	4.88 ± 1.98	1.052	[37]
	Control—49	35.12 ± 4.37	2.76 ± 2.04		
Cirik (2016) Turkey	Study group—101	24.44 ± 4.23	7.0 ± 4.0	0.286	[45]
	Control—49	26.29 ± 5.17	6.0 ± 2.0		
Sukhapure (2022) New Zealand	Study group—33	29.24 ± 6.81	4.82 ± 4.0	0.492	[47]
	Control—41	29.29 ± 8.62	3.0 ± 3.36		
Dag (2017) Turkey	Study group—53	22.69 ± 4.54	5.47 ± 2.97	0.070	[49]
	Control—38	21.34 ± 2.12	5.23 ± 3.96		

Pooled SMD (Hedge’s g): 0.421, I2 = 67%; Q = 15.30, *p* = 0.01.

**Table 6:** Probability of depression in PCOS patients.

Study (Year)/Country	PCOS (Active/Cases)	Control (Active/Cases)	RR [95%CI]	Citation
Karsten (2021)/UK	47.9% (35/73)	47% (47/100)	1.02 [0.74–1.40]	[7]
Dybciak (2022)/Poland	41.7% (96/230)	16.1% (32/199)	2.60 [1.83–3.69]	[16]
Tariq (2021)/Pakistan	55.6% (75/135)	8.9% (12/135)	6.25 [3.57–10.95]	[20]
Batool (2016)/Pakistan	30% (41/137)	9% (12/137)	3.42 [1.88–6.214]	[35]
Cirik (2016)/Turkey	46.5% (47/101)	20.4% (10/49)	2.28 [1.26–4.12]	[45]

Pooled RR: 2.58 [1.38–4.85]; I^2^ = 90%, Q = 38.45 *p* < 0.001.

## Data Availability

Not applicable.

## References

[1] Bozdag G., Mumusoglu S., Zengin D., Karabulut E., Yildiz B.O. (2016). The prevalence and phenotypic features of polycystic ovary syndrome: A systematic review and meta-analysis. Hum. Reprod..

[2] Lizneva D., Suturina L., Walker W., Brakta S., Gavrilova-Jordan L., Azziz R. (2016). Criteria, prevalence, and phenotypes of polycystic ovary syndrome. Fertil. Steril..

[3] Nidhi R., Padmalatha V., Nagarathna R., Amritanshu R. (2011). Prevalence of Polycystic Ovarian Syndrome in Indian Adolescents. J. Pediatr. Adolesc. Gynecol..

[4] Christ J.P., Cedars M.I. (2023). Current Guidelines for Diagnosing PCOS. Diagnostics.

[5] Fauser B.C., Tarlatzis B.C., Rebar R.W., Legro R.S., Balen A.H., Lobo R., Carmina E., Chang J., Yildiz B.O., Laven J.S. (2012). Consensus on women’s health aspects of polycystic ovary syndrome (PCOS): The Amsterdam ESHRE/ASRM-Sponsored 3rd PCOS Consensus Workshop Group. Fertil. Steril..

[6] Çetinkaya A.S., Çelik Ö., Özer Ü., Çolak S. (2022). Depression, anxiety, body image scores, and sexual dysfunction in patients with polycystic ovary syndrome according to phenotypes. Gynecol. Endocrinol..

[7] Karsten M.D.A., Wekker V., Groen H., Painter R.C., Mol B.W.J., Laan E.T.M., Roseboom T.J., Hoek A. (2021). The role of PCOS in mental health and sexual function in women with obesity and a history of infertility. Hum. Reprod. Open..

[8] Ali A.T. (2015). Polycystic ovary syndrome and metabolic syndrome. Ceska Gynekol..

[9] Borghi L., Leone D., Vegni E., Galiano V., Lepadatu C., Sulpizio P., Garzia E. (2018). Psychological distress, anger and quality of life in polycystic ovary syndrome: Associations with biochemical, phenotypicalandsocio-demographic factors. J. Psychosom. Obstet. Gynecol..

[10] Papalou O., Diamanti-Kandarakis E. (2017). The role of stress in PCOS. Expert Rev. Endocrinol. Metab..

[11] Gholinezhad M., Golsorkhtabaramiri M., Esmaeilzadeh S., Ghanbarpour A. (2018). Insulin resistance and adverse metabolic profile in overweight/obese and normal weight of young women with polycystic ovary syndrome. Casp. J. Intern. Med..

[12] Legro R.S., Arslanian S.A., Ehrmann D.A., Hoeger K.M., Murad M.H., Pasquali R., Welt C.K. (2013). Diagnosis and Treatment of Polycystic Ovary Syndrome: An Endocrine Society Clinical Practice Guideline. J. Clin. Endocr..

[13] Almeshari W.K., Alsubaie A.K., Alanazi R.I., Almalki Y.A., Masud N., Mahmoud S.H. (2021). Depressive and Anxiety Symptom Assessment in Adults with Polycystic Ovarian Syndrome. Depress. Res. Treat..

[14] Brutocao C., Zaiem F., Alsawas M., Morrow A.S., Murad M.H., Javed A. (2018). Psychiatric disorders in women with polycystic ovary syndrome: A systematic review and meta-analysis. Endocrine.

[15] Deeks A.A., Gibson-Helm M.E., Teede H.J. (2010). Anxiety and depression in polycystic ovary syndrome: A comprehensive investigation. Fertil. Steril..

[16] Dybciak P., Humeniuk E., Raczkiewicz D., Krakowiak J., Wdowiak A., Bojar I. (2022). Anxiety and Depression in Women with Polycystic Ovary Syndrome. Medicina.

[17] Sheikh J., Hebbar M., Zia N., Wicks S., Jayaprakash S., Narendran A., Khalil H., Melson E., Busby M., Tahrani A. (2021). Increased anxiety, depression and body dysmorphia in women with polycystic ovary syndrome: Results from blue morpho survey. Endocr. Abstr..

[18] Zehravi M., Maqbool M., Ara I. (2021). Depression and anxiety in women with polycystic ovarian syndrome: A literature survey. Int. J. Adolesc. Med. Health.

[19] Radhakrishnan R., Verghese A. (2018). A study on anxiety and depression among patients with polycystic ovary syndrome. JDDT.

[20] Tariq A., Tariq M.M., Tariq H., Khattak S.N., Yazdani T., Malik R.T., Rauf S., Malik T. (2021). Frequency and Risk Factors for Depression and Anxiety in Patients with Polycystic Ovary Syndrome Presenting in a Tertiary Care Hospital Karachi, Pakistan. J. Obstet. Gynaecol..

[21] Mansson M., Holte J., Landin-Wilhelmsen K., Dahlgren E., Johansson A., Landén M. (2008). Women with polycystic ovary syndrome are often depressed or anxious-A case control study. Psychneuroendocrinology.

[22] Vos T., Allen C., Arora M., Barber R.M., Bhutta Z.A., Brown A., Carter A., Casey D.C., Charlson F.J., Chen A.Z. (2016). Global, regional, and national incidence, prevalence, and years lived with disability for 310 diseases and injuries, 1990-2015: A systematic analysis for the Global Burden of Disease Study 2015. Lancet.

[23] Sadock B.J., Sadock V.A. (2007). Mood disorders. Kaplan & Sadock’s Synopsis of Psychiatry. Behavioral Sciences/Clinical Psychiatry.

[24] Kowalczyk M., Kowalczyk E., Galita G., Majsterek I., Talarowska M., Popławski T., Kwiatkowski P., Lichota A., Sienkiewicz M. (2022). Association of Polymorphic Variants in Argonaute Genes with Depression Risk in a Polish Population. Int. J. Mol. Sci..

[25] Gałecki P., Talarowska M. (2018). Inflammatorytheory of depression. Psychiatr. Pol..

[26] Zheng K., Chu J., Zhang X., Ding Z., Song Q., Liu Z., Peng W., Cao W., Zou T., Yi J. (2022). Psychologicalresilience and dailystressmediate the effect of childhood trauma on depression. Child Abus. Negl..

[27] Accortt E.E., Freeman M.P., Allen J.J.B. (2008). Women and Major Depressive Disorder: Clinical Perspectives on Causal Pathways. J. Womens Health.

[28] Blay S.L., Aguiar J., Passos I.C. (2016). Polycystic ovary syndrome and mental disorders: A systematic review and exploratory meta-analysis. Neuropsychiatr. Dis. Treat..

[29] Cooney L.G., Lee I., Sammel M.D., Dokras A. (2017). High prevalence of moderate and severe depressive and anxiety symptoms in polycystic ovary syndrome: A systematic review and meta-analysis. Hum. Reprod..

[30] Dokras A., Clifton S., Futterweit W., Wild R. (2011). Increased Risk for Abnormal Depression Scores in Women with Polycystic Ovary Syndrome: A Systematic Review and Meta-Analysis. Obstet. Gynecol..

[31] Wang Y., Ni Z., Li K. (2021). The prevalence of anxiety and depression of different severity in women with polycystic ovary syndrome: A meta-analysis. Gynecol. Endocrinol..

[32] Yin X., Ji Y., Chan C.L., Chan C.H.Y. (2021). The mental health of women with polycystic ovary syndrome: A systematic review and meta-analysis. Arch. Womens Ment. Health.

[33] Kogure G.S., Ribeiro V.B., Lopes I.P., Furtado C.L.M., Kodato S., de Sá M.F.S., Ferriani R.A., Lara L.A.d.S., dos Reis R.M. (2019). Body image and its relationships with sexual functioning, anxiety, and depression in women with polycystic ovary syndrome. J. Affect. Disord..

[34] Bazarganipour F., Ziaei S., Montazeri A., Foroozanfard F., Kazemnejad A., Faghihzadeh S. (2013). Psychological investigation in patients with polycystic ovary syndrome. Health Qual. Life Outcomes.

[35] Batool S., Ul Ain Ahmed F., Ambreen A., Sheikh A., Faryad N. (2016). Depression and Anxiety in Women with Polycystic Ovary Syndrome and Its Biochemical Associates. JSAFOG.

[36] Xing L., Xu J., Wei Y., Chen Y., Zhuang H., Tang W., Yu S., Zhang J., Yin G., Wang R. (2022). Depression in polycystic ovary syndrome: Focusing on pathogenesis and treatment. Front. Psychiatry.

[37] Barry J.A., Kuczmierczyk A.R., Hardiman P.J. (2011). Anxiety and depression in polycystic ovary syndrome: A systematic review and meta-analysis. Hum. Reprod..

[38] Bjelland I., Dahl A.A., Haug T.T., Neckelmann D. (2002). The validity of the Hospital Anxiety and Depression Scale. J. Psychosom. Res..

[39] Snaith R.P. (2003). The Hospital Anxiety And Depression Scale. Health Qual. Life Outcomes.

[40] Moher D., Liberati A., Tetzlaff J., Altman D.G., PRISMA Group (2009). Preferred reporting items for systematic reviews and meta-analyses: The PRISMA statement. PLoS Med..

[41] Lujan M.E., Chizen D.R., Pierson R.A. (2008). Diagnostic Criteria for Polycystic Ovary Syndrome: Pitfalls and Controversies. J. Obstet. Gynaecol. Can..

[42] Saxena R., Singh P., Verma A., Sharma M. (2022). Relationship between anxiety, depression and quality of life in medical student with polycystic ovary syndrome. Int. J. Reprod. Contracept. Obstet. Gynecol..

[43] Alur-Gupta S., Lee I., Chemerinski A., Liu C., Lipson J., Allison K., Gallop R., Dokras A. (2021). Racial differences in anxiety, depression, and quality of life in women with polycystic ovary syndrome. F&S Rep..

[44] Light R.S., Chilcot J., McBride E. (2020). Psychological Distress in Women Living with Polycystic Ovary Syndrome: The Role of Illness Perceptions. Women’s Health Issues.

[45] Akdağ Cirik D., Dilbaz B., Aksakal S., Kotan Z., Özelçi R., Akpinar F., Mollamahmutoğlu L. (2016). Do anxiety and depression statuses differ in differentpolycystic ovary syndrome phenotypes? Turk. J. Med. Sci..

[46] Benson S., Hahn S., Tan S., Mann K., Janssen O., Schedlowski M., Elsenbruch S. (2009). Prevalence and implications of anxiety in polycystic ovary syndrome: Results of an internet-based survey in Germany. Hum. Reprod..

[47] Sukhapure M., Eggleston K., Fenton A., Frampton C., Porter R.J., Douglas K.M. (2022). Changes in Mood, Anxiety, and Cognition with Polycystic Ovary Syndrome Treatment: A Longitudinal, Naturalistic Study. Neuropsychiatr. Dis. Treat..

[48] Fatemeh B., Shahideh J.S., Negin M. (2021). Health related quality of life and psychological parameters in different polycystic ovary syndrome phenotypes: A comparative cross-sectional study. J. Ovarian Res..

[49] Dag O., Alpua M., Isik Y., Buturak S.V., Tulmac O.B., Turkel Y. (2017). The evaluation of temperament and quality of life in patients with polycystic ovary syndrome. Gynecol. Endocrinol..

[50] Veltman-Verhulst S.M., Boivin J., Eijkemans M.J.C., Fauser B.J. (2012). Emotional distress is a common risk in women with polycystic ovary syndrome: A systematic review and meta-analysis of 28 studies. Hum. Reprod. Update.

[51] Sadeeqa S., Mustafa T., Latif S. (2018). Polycystic Ovarian Syndrome-Related Depression in Adolescent Girls: A Review. J. Pharm. Bioallied Sci..

[52] Fekadu N., Shibeshi W., Engidawork E. (2017). Major depressivedisorder: Pathophysiology and clinical management. J. Depress. Anxiety.

[53] Miller A.H., Haroon E., Felger J.C. (2017). TherapeuticImplications of Brain-ImmuneInteractions: Treatment in Translation. Neuropsychopharmacology.

[54] Escobar-Morreale H.F., Luque-Ramírez M., González F. (2011). Circulating inflammatory markers in polycysticovary syndrome: A systematic review and meta analysis. Fertil. Steril..

[55] Nathan R.S., Sachar E.J., Asnis G.M., Halbreich U., Halpern F.S. (1981). Relative insulin insensitivity and cortisolsecretion in depressedpatients. Psychiatry Res..

[56] Kolhe J.V., Chhipa A.S., Butani S., Chavda V., Patel S.S. (2022). PCOS and Depression: Common Links and Potential Targets. Reprod. Sci..

[57] Siu A.L., US Preventive Services Task Force (USPSTF) (2016). Screening for Depression in Adults: US Preventive Services Task Force Recommendation Statement. JAMA.

[58] Teede H., Gibson-Helm M., Norman R.J., Boyle J. (2014). Polycystic Ovary Syndrome: Perceptions and Attitudes of Women and Primary Health Care Physicians on Features of PCOS and Renaming the Syndrome. J. Clin. Endocr..

[59] Colwell K., Lujan M.E., Lawson K.L., Pierson R.A., Chizen D.R. (2010). Women’s Perceptions of Polycystic Ovary Syndrome Following Participation in a Clinical Research Study: Implications for Knowledge, Feelings, and Daily Health Practices. J. Obstet. Gynaecol. Can..

[60] Shrivastava Y., Jagdev P. (2019). A Study to assess the Effectiveness of self Instructional module on Knowledge regarding Polycystic Ovarian Syndrome among B. Sc. Nursing students of Selected nursing college. Asian J. Nurs. Educ. Res..

[61] Abdollahi L., Mirghafourvand M., Babapour J.K., Mohammadi M. (2019). Effectiveness of cognitive-behavioral therapy (CBT) in improving the quality of life and psychological fatigue in women with polycystic ovarian syndrome: A randomized controlled clinical trial. J. Psychosom. Obstet. Gynaecol..

